# Protein engineering and other bio-synthetic routes for bio-based materials: current uses and potential applications

**DOI:** 10.3389/fchem.2014.00083

**Published:** 2014-10-13

**Authors:** Carissa M. Soto

**Affiliations:** U. S. Naval Research Laboratory, Center for Bio/Molecular Science and EngineeringWashington, DC, USA

**Keywords:** hydrogels, biocatalysis, human cholinesterase, bacterial collagen, biomass degradation

Since the pioneer work of Stanley Cohen, Herbert Boyer and their collaborators almost 40 years ago (Cohen et al., 1973), genetic engineering had advanced greatly. The tools and resources available to the genetic engineer open new opportunities in genome engineering (Carr and Church, 2009) and synthetic biology (Lu et al., 2009). But still much needs to be done to build and control biological systems. In the current Research Topic, we focus on the synthesis of bio-based materials employing the basic principles of genetic engineering. The rationale of utilizing biosynthesis as a route for material production relies on the capability of the biological machinery to produce macromolecules of well-defined sequence, stereochemistry, and size. The specific location of the chemical groups in a given bio-macromolecule is critical to its structure and ultimately its biological function. Protein engineering has emerged as a strong tool to produce proteins in large scale with high fidelity. In the context of the current Research Topic we refer to such protein products as bio/macromolecules, nanostructures, biologicals or precursors produced in a bio-based environment. The topic is designed to present several approaches to biosynthesis, not limited to the production of a target protein in a bacterial host.

At first, I would like to direct the readers to an opinion article that presents a broad perspective of the field of bio-based and bio-inspired materials (Nick McElhinny and Becker, 2014). Following their theme, most of the articles in the current Research Topic fit in the category of *Using Biology as a Material*. For example, several original research papers report the utilization of genetically-engineered proteins to obtain materials for various applications such as: tunable hydrogels (Tang and Olsen, 2014), hydrogels for mechanically active tissues (Li and Kiick, 2014), bacterial collagen for tissue engineering (An et al., 2014), and microfibers for nerve guide conduits (Kakinoki et al., 2014). Plant viruses (Pongkwan et al., 2014) and antimicrobial peptides (Rodriguez et al., 2014) are the starting bio-based materials to incorporate new functionalities by well-known chemical methods. In the field of biocatalysis, immobilized enzymes are used for industry-scale production of bioproducts (López et al., 2014) and bacterial homologs of human cholinesterase (Legler et al., 2014) are engineered in the laboratory setting with the aim of using them as a therapeutic enzyme.

From the materials science perspective, one can easily envision using a biological organism guided by well-established biotechnology techniques to generate a single product. However, the biological environment still has more to offer. An example is described in the review article where *Clostridium thermocellum* is presented as a strong candidate in bioprocessing applications. The system attributes are due to its unique, multivariate enzyme cellulosome complex and its role during biomass degradation (Akinosho et al., 2014). Studies of complex cellular processes without disturbing natural biological events (Chambers, 2014) are needed to expand our knowledge and ultimately to facilitate the engineering of biological systems for our benefit. Lastly, a new paradigm is presented in which bio-building blocks and non-standard amino acids are utilized in a cell-free environment (Hong et al., 2014). While others incorporate artificial amino acids into protein products using cell-based biosynthesis strategies, having a cell-free environment offers alternatives to new applications where a cell-free environment is more desirable. We can imagine using a cell-free environment for the biosynthesis of more complex systems where biomacromolecules are produced in a concerted way to control synthesis and/or assembly.

There is no doubt that bio-based products provide a rich tool-box with a great potential for a wide variety of applications as presented in the current Research Topic. Taking a multidisciplinary approach, combining knowledge from biology, chemistry, and materials science to produce complex materials is crucial to succeed in this field. Still challenges exist for the utilization of bio-based materials in main stream industrial production. Public acceptance and cost effectiveness will be key elements in engaging the industry into adapting these new biosynthetic strategies as alternatives to classical chemical synthetic routes. For example, one general public concern on using plants to produce materials is the limitation in arable land and fresh water which are needed to fulfill future demands on food for a constantly increasing population (Ronald, 2011). Furthermore, genetic engineering can be of a great concern if the engineered systems are delivered to the environment causing a spread of un-desirable traits or antibiotic resistant bacterial strains. In spite of such concerns, major advances in the production of biopolymers at the industrial scale are already implemented by major bioplastics producers (Smith, 2012). Proper engineer controls must be implemented to avoid *biological contamination* similarly as chemicals production is tightly regulated. As the field advances and we increase our understanding of biological synthetic platforms and our ability to engineer those systems improves, we should be able to generate materials previously not imaginable.

## Conflict of interest statement

The author declares that the research was conducted in the absence of any commercial or financial relationships that could be construed as a potential conflict of interest.
